# Relationship between nocturia, depression, and cognitive function and the mediating effects of nutritional indexes in older adults: data from NHANES 2011–2014

**DOI:** 10.3389/fnut.2025.1533683

**Published:** 2025-07-02

**Authors:** Yin Xu, Xinmei Wang, Guofeng Wang, Wei Wei, Ning Li

**Affiliations:** ^1^Department of Gerontology, The 960th Hospital of PLA, Jinan, China; ^2^The Academy of Military Medical Sciences of China, Beijing, China

**Keywords:** nocturia, cognitive function, depression, older adults, albumin, hemoglobin

## Abstract

**Purpose:**

This study evaluated the correlation between nocturia, depression, and cognitive function in older adults and the mediating effect of albumin and hemoglobin on this correlation.

**Methods:**

Data on nocturia, depression, and cognitive function from the National Health and Nutrition Examination Survey 2011–2014 were analyzed by multiple logistic regression.

**Results:**

The digit symbol score (DSS) and 9-Item Patient Health Questionnaire (PHQ-9) scores were linearly and non-linearly correlated with nocturia risk (*p* < 0.05). Male/female-stratified analysis showed that animal fluency scores (AFS), DSS, and PHQ-9 scores were significantly correlated with the risk of nocturia in females (*p* < 0.05), and PHQ-9 scores were significantly associated with the risk of nocturia in males (*p* < 0.05). Albumin partially mediated the association of AFS, DSS, and PHQ-9 scores with nocturia risk in women and the relationship of PHQ-9 scores with nocturia risk in men. Hemoglobin partially mediated the relationship of AFS and DSS with nocturia risk in women and the association of PHQ-9 scores with nocturia risk in men.

**Conclusion:**

Nocturia is positively associated with depression and cognitive impairment in older adults, especially in women. Nutrition partially mediates the relationship between nocturia, depression, and cognitive function. Thus, improving nutrition may decrease the risk of nocturia in older adults.

## 1 Introduction

Nocturia, defined as waking at night to void, is a prevalent condition affecting a significant proportion of the adult population and is increasingly recognized as a multifaceted syndrome with systemic health implications (1–4). Emerging evidence underscores bidirectional associations between nocturia and neuropsychiatric disorders, particularly depression and cognitive dysfunction, mediated through interconnected pathophysiological pathways. For instance, depression severity demonstrates a dose-dependent relationship with nocturia, with studies indicating heightened depression risk in individuals affected by nocturia (5, 6). Similarly, cognitive dysfunction is also linked to nocturia, as systematic reviews report significant associations between the two conditions, though causal inferences are limited by the predominance of cross-sectional designs (4).

The underlying mechanisms involve disruptions in the hypothalamic-pituitary-adrenal (HPA) axis, circadian rhythm disturbances, and inflammatory cascades. HPA axis hyperactivity, often observed in depression, elevates the secretion of pro-inflammatory cytokines such as IL-6 and TNF-α, impairing neuroplasticity and contributing to neuropsychiatric symptoms (7, 8). Sleep fragmentation, a common feature of nocturia, exacerbates HPA axis dysregulation and reduces amyloid-beta clearance efficiency, thereby accelerating cognitive decline (9, 10). Additionally, circadian misalignment and alterations in the tryptophan-kynurenine pathway contribute to neurotoxicity through excitotoxicity and oxidative stress, with dysregulated metabolites implicated in depressive pathology (11–14).

Nutritional status acts as a critical modulator in this network, with aberrant expression of serum albumin (Alb) and hemoglobin exacerbating neuropsychiatric outcomes. Specifically, reduced Alb and hemoglobin levels are associated with increased inflammation and oxidative stress, which amplify neurotoxic cascades and compromise blood-brain barrier function (13, 15, 16). Hypoalbuminemia and anemia may also impair neuroprotective pathways, while imbalances in the kynurenine pathway elevate quinolinic acid concentrations, leading to NMDA receptor-mediated excitotoxicity (12–14). Causal mediation analyses suggest nutritional biomarkers contribute up to 21.9% of associations between muscle-related factors and depressive symptoms, highlighting their potential as mechanistic bridges (13, 16).

Despite these advances, critical knowledge gaps persist. Current research relies heavily on cross-sectional data, precluding causal inference and limiting the exploration of temporal dynamics (4, 17). Non-linear dose-response relationships between nocturia severity, depression, and cognitive dysfunction remain underexplored, and sex-specific pathophysiological differences (e.g., gender disparities in biomarker profiles) are inadequately characterized (6). Furthermore, the role of nutritional mediators, such as serum Alb and hemoglobin, in bridging urinary dysfunction with neuropsychiatric outcomes requires deeper investigation through advanced methodologies like restricted cubic splines (RCS) and formal causal mediation analysis (4, 13). To address these gaps, this study employs a comprehensive analytical framework integrating multivariate logistic regression, RCS for modeling non-linear effects, and causal mediation analysis. Building on prior epidemiological findings (4, 9), we aim to: (1) quantify independent associations between nocturia, depression severity, and cognitive dysfunction in at-risk populations; (2) characterize non-linear dose-response patterns using RCS, adjusted for demographic and behavioral confounders; (3) evaluate the mediating roles of serum Alb and hemoglobin in these associations; and (4) investigate sex- and age-stratified effect modifications. Our findings are expected to elucidate nutritional biomarkers as pivotal factors in the nocturia-neuropsychiatric nexus, informing targeted interventions for vulnerable cohorts.

## 2 Methods

### 2.1 Study population

**Figure 1:**
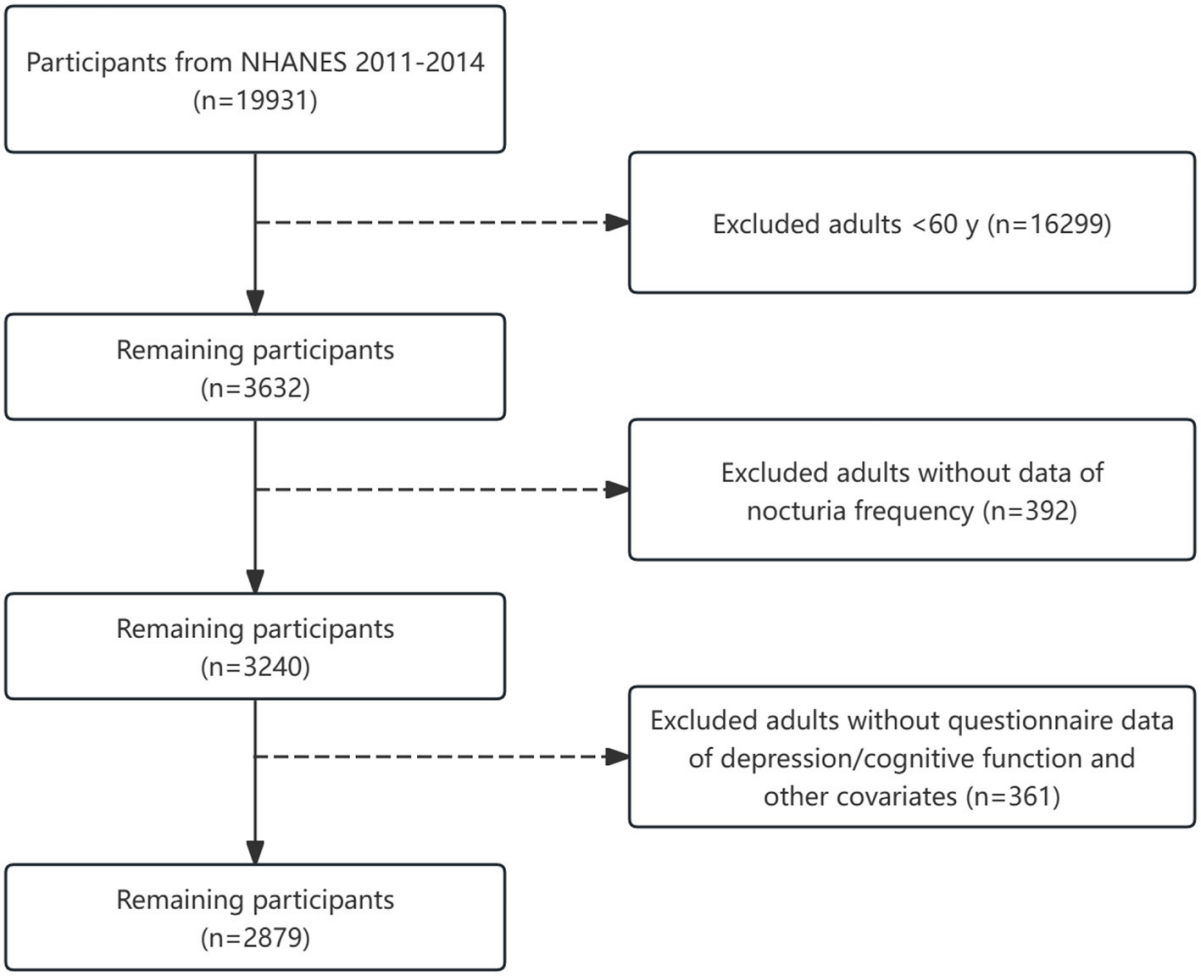
The flowchart of patient selection.

This study analyzed data from National Health and Nutrition Examination Survey (NHANES) 2011–2014. A detailed description of the data collection method can be found on the NHANES website (https://wwwn.cdc.gov/nchs/nhanes/Default.aspx). Two data cycles (2011–2014) involving 2,879 participants (48.6% males and 51.4% females) were analyzed. The exclusion criteria were participants younger than 60 years and participants with missing data on nocturia frequency, blood test results, body mass index, arterial blood pressure, glycosylated HGB levels, physical activity, smoking status, and drinking status. The flowchart of patient selection is shown in Figure 1.

### 2.2 Nocturia

Data on the number of times the participants woke up at night to void were obtained by trained interviewers at the Mobile Examination Center (MEC) using the CAPI system. Nocturia was defined as at least two voids per night. Details on the administration of the questionnaire can be found in the MEC Interviewer Exam Manual.

### 2.3 Depression and cognitive function

The frequency of depressive symptoms over the past 2 weeks was measured using the 9-item Patient Health Questionnaire (PHQ-9). Cognitive function was assessed using word learning and recall modules, the animal fluency test, and the digit symbol substitution test (DSST). The CERAD word learning subtest assesses the immediate and delayed ability to learn new verbal information (memory subdomain). The test consists of three consecutive learning trials and a delayed recall. The immediate recall score (IRS) was calculated by summing the scores of three successive trials. The total word recall score (TWRS) was calculated by summing IRS and the delayed word recall score (DWRS). In order to reduce Category 1 errors, we selected total score (TWRS) instead of sub-score (IRS and DWRS) to study the correlation between nocturia and cognition (18, 19). The animal fluency test examined language fluency by asking the participants to name as many animals as possible in 1 min. The score was recorded as the animal fluency score (AFS). The DSST is a module of the Wechsler Adult Intelligence Scale that assesses processing speed, sustained attention, and working memory. In the DSST, participants are asked to substitute each number with a symbol within 120 s. The maximum possible score is 133 points. The digit symbol score (DSS) was the total number of correct answers. The cognitive function score was standardized into *Z*-score, which served as a metric for classifying the level of cognitive function (20). *Z*-score ≤ −1.5 SD is generally considered the threshold for mild cognitive impairment (MCI), while the normal range is defined as *Z*-score ≥ −1.0 SD (21, 22).

### 2.4 Nutritional index

Blood counts and biochemical tests were used to measure serum ALB and glycosylated HGB, respectively. Blood samples were collected, processed, frozen at −20°C, and sent to the Collaborative Laboratory Services for analysis. Comprehensive guidelines on specimen collection and processing are available in the NHANES Laboratory Procedures Manual.

### 2.5 Covariates

All covariates were selected through single-factor analysis (23–26). Angina, congestive heart failure, coronary heart disease, type 2 diabetes mellitus, or stroke were considered chronic diseases. The participants were categorized into three groups based on smoking status: never smokers, former smokers, and current smokers. Never smokers were individuals who had never smoked or had smoked fewer than 100 cigarettes in their lifetime. Former smokers were those who had smoked at least 100 cigarettes but quit smoking (27). Drinkers were defined as individuals who consumed at least 12 drinks in 12 months. Blood pressure was measured consecutively three times by a medical professional using a sphygmomanometer, and the readings were averaged. Hypertension was defined as average systolic blood pressure ≥140 mmHg or diastolic blood pressure ≥90 mmHg. T2DM was defined by being diagnosed by a health professional or having HbA1c levels of ≥6.5% or fasting glucose ≥7.0 mmol/L (28).

### 2.6 Statistical analyses

Data were weighted according to NHANES requirements to be representative of the general population in the United States. The NHANES 2011–2014 data modules utilized in this study were sourced from the official public database of the Centers for Disease Control and Prevention (CDC). The data ownership resides with the United States Department of Health and Human Services (HHS). Researchers acquire these data by adhering to the standard data usage protocol, without requiring additional authorization. All analyses were performed using EmpowerStats statistical software (X&Y Solutions, Boston, MA) and R software version 4.3.2. In this study, multiple interpolation filling was performed on the missing values. After interpolation, the data integrity was examined, respectively, the distribution before and after interpolation was compared, the rationality of the interpolation values was checked, and the sensitivity analysis of different interpolation methods (PMM, random forest, Bayesian regression) and the diagnosis of interpolation models were conducted. Subsequent analysis is conducted using the interpolated data. Continuous variables were expressed as median (P25, P75), and categorical variables were expressed as percentages. If the variable was continuous, the Kruskal–Wallis rank-sum test was employed. For counting variables with a theoretical value < 10, the Fisher's exact probability test was utilized. The analysis process was done by Empowerstats. The participants were divided into two groups based on the presence or absence of nocturia. The relationship between nocturia, depression, and cognitive function was evaluated using multivariate logistic regression analysis and restricted cubic splines. We adjusted for covariates and performed multiple regression analyses. The results were stratified by male/female, and sensitivity analysis was performed. The mediating effect of ALB and HGB on the association between nocturia, depression, and cognitive function was assessed using the mediation package in R (Bootstrap = 5,000, prop. mediated = ACME (average)/total effect) (29). All RCS models were adjusted for male/female, age, race, education level, marital status, PIR, BMI, hypertension, diabetes, smoking status, alcohol drinking status, work activities, and recreational activities as covariates. Subgroup analyses of male/female and age were used to study the stability and specificity of mediating effects. The Akaike Information Criterion (AIC) value was used to determine the optimal number of knots, the number of knots was three for DWRS (10, 50, and 90th percentiles) and PHQ-9 Score, and five for AFS and DSS (5, 25, 50, 75 and 95th percentiles). If the indirect effect was not significant (*p* > 0.05), the intermediate ratio was not calculated (30). In the sensitivity analysis, the correlation between cognitive function and nocturia was examined using multiple regression analysis after adjustment for confounding factors. *p*-Values of < 0.05 were considered statistically significant.

## 3 Results

### 3.1 Participant characteristics

The study included 2,879 participants with a mean age of 69.44 ± 6.77 years. The demographic and clinical characteristics of the study population are shown in Table 1. Nocturia was associated with older age, Mexican American or non-Hispanic Black ethnicity, lower education, unmarried status, higher body mass index, lower poverty-to-income ratio, alcohol drinking, current smoking, higher engagement in work or recreational activities, and higher prevalence of diabetes and hypertension. Furthermore, nocturia was linked with lower DWRS, AFS, and DSS and higher PHQ-9 scores (*p* < 0.05).

**Table 1:** Demographic characteristics of the cohort.

**Characteristic**	**No nocturia**	**Nocturia**	***p*-Value**
*N*	1,555	1,324	
Age [years, median (IQR)]	68.00 (63.00-74.00)	69.00 (64.00-76.00)	<0.001
**Male/female (%)**			**0.592**
Male	749 (48.17%)	651 (49.17%)	
Female	806 (51.83%)	673 (50.83%)	
**Race/ethnicity (%)**			<**0.001**
Mexican American	122 (7.85%)	128 (9.67%)	
Other Hispanic	154 (9.90%)	141 (10.65%)	
Non-Hispanic White	836 (53.76%)	542 (40.94%)	
Non-Hispanic Black	295 (18.97%)	385 (29.08%)	
Other race	148 (9.52%)	128 (9.67%)	
**Education level (%)**			<**0.001**
Less than high school	309 (19.87%)	414 (31.34%)	
High school graduate/GED or equivalent	347 (22.32%)	326 (24.68%)	
College or above	899 (57.81%)	581 (43.98%)	
**Marital status (%)**			<**0.001**
Married/living as married	954 (61.39%)	708 (53.60%)	
Single/divorced/widowed/never married	600 (38.61%)	613 (46.40%)	
Body mass index [kg/m^2^, median (IQR)]	27.60 (24.30–31.40)	28.60 (25.00–33.20)	<0.001
PIR [median (IQR)]	2.59 (1.33–4.92)	1.88 (1.04–3.38)	<0.001
**Drinking status (%)**			**0.003**
No	458 (29.47%)	458 (34.64%)	
Yes	1,096 (70.53%)	864 (65.36%)	
**Smoking status (%)**			**0.884**
No	761 (48.97%)	655 (49.51%)	
Now	591 (38.03%)	504 (38.10%)	
Former	202 (13.00%)	164 (12.40%)	
**Work activity (%)**			<**0.001**
Vigorous	188 (12.10%)	119 (9.00%)	
Moderate	332 (21.36%)	229 (17.32%)	
Other	1,034 (66.54%)	974 (73.68%)	
**Recreational activity (%)**			<**0.001**
Vigorous	179 (11.51%)	89 (6.72%)	
Moderate	534 (34.34%)	402 (30.36%)	
Other	842 (54.15%)	833 (62.92%)	
**Hypertension (%)**			<**0.001**
No	517 (34.33%)	300 (23.27%)	
Yes	989 (65.67%)	989 (76.73%)	
**Diabetes (%)**			<**0.001**
No	1,196 (77.06%)	905 (68.46%)	
Yes	356 (22.94%)	417 (31.54%)	
Immediate recall score [median (IQR)]	20.00 (16.00–23.00)	19.00 (15.00–22.00)	<0.001
Delayed word recall score [median (IQR)]	6.00 (5.00–8.00)	6.00 (4.00–7.00)	<0.001
Total word recall [median (IQR)]	26.00 (21.00–30.00)	25.00 (20.00–29.00)	<0.001
Animal fluency score [median (IQR)]	17.00 (13.00–21.00)	16.00 (12.00–19.00)	<0.001
Digit symbol score [median (IQR)]	49.00 (37.00–61.00)	42.00 (30.00–54.00)	<0.001
PHQ-9 score [median (IQR)]	1.00 (0.00–3.00)	2.00 (0.00–6.00)	<0.001
Hemoglobin [g/L, median (IQR)]	13.90 (13.10–14.80)	13.60 (12.60–14.50)	<0.001
Albumin [g/L, median (IQR)]	4.20 (4.00–4.40)	4.20 (4.00–4.40)	<0.001

Data are median (P25, P75) or numbers and percentages.

p-Value: Kruskal–Wallis rank sum test for continuous variables, Fisher exact test for categorical variables with expects < 10.

### 3.2 Correlation between nocturia, depression, and cognitive function

Multiple regression analysis revealed that TWRS, AFS, DSS, and PHQ-9 scores were significantly associated with the risk of nocturia. In the unadjusted model, higher cognitive function scores (TWRS, AFS, and DSS) were associated with a lower risk of nocturia, and higher PHQ-9 scores were linked with a higher risk of nocturia. After adjusting for variables, the association of DSS and PHQ-9 scores with the risk of depression remained significant (Table 2).

**Table 2:** Multiple regression analysis of the association of cognitive function and depression scores with the risk of nocturia in older adults.

**Exposure**	**Non-adjusted**	**Model I**	**Model II**
Immediate recall scores	0.9534 (0.9382, 0.9688), *p* < 0.000001	0.9821 (0.9638, 1.0008), *p* = 0.060652	0.9819 (0.9628, 1.0015), *p* = 0.069880
Delayed word recall scores	0.9398 (0.9104, 0.9701), *p* = 0.000126	1.0068 (0.9697, 1.0453), *p* = 0.722962	1.0028 (0.9642, 1.0430), *p* = 0.887868
Total word recall scores	0.9686 (0.9576, 0.9796), *p* < 0.000001	0.9916 (0.9782, 1.0051), *p* = 0.221534	0.9910 (0.9770, 1.0051), *p* = 0.209903
Animal fluency scores	0.9469 (0.9338, 0.9602), *p* < 0.000001	0.9827 (0.9667, 0.9990), *p* = 0.037828	0.9853 (0.9684, 1.0025), *p* = 0.092566
Digit symbol scores	0.9752 (0.9708, 0.9796), *p* < 0.000001	0.9876 (0.9814, 0.9939), *p* = 0.000108	0.9890 (0.9824, 0.9958), *p* = 0.001430
9-item patient health questionnaire scores	1.0879 (1.0684, 1.1078), *p* < 0.000001	1.0823 (1.0606, 1.1043), *p* < 0.000001	1.0749 (1.0525, 1.0978), *p* < 0.000001

Model I was adjusted for male/female, age, race, education level, marital status, and poverty-to-income ratio.

Model II was adjusted for male/female, age, race, education level, marital status, and poverty-to-income ratio, body mass index, hypertension, diabetes, smoking status, alcohol drinking status, work activities, and recreational activities.

**Figure 2:**
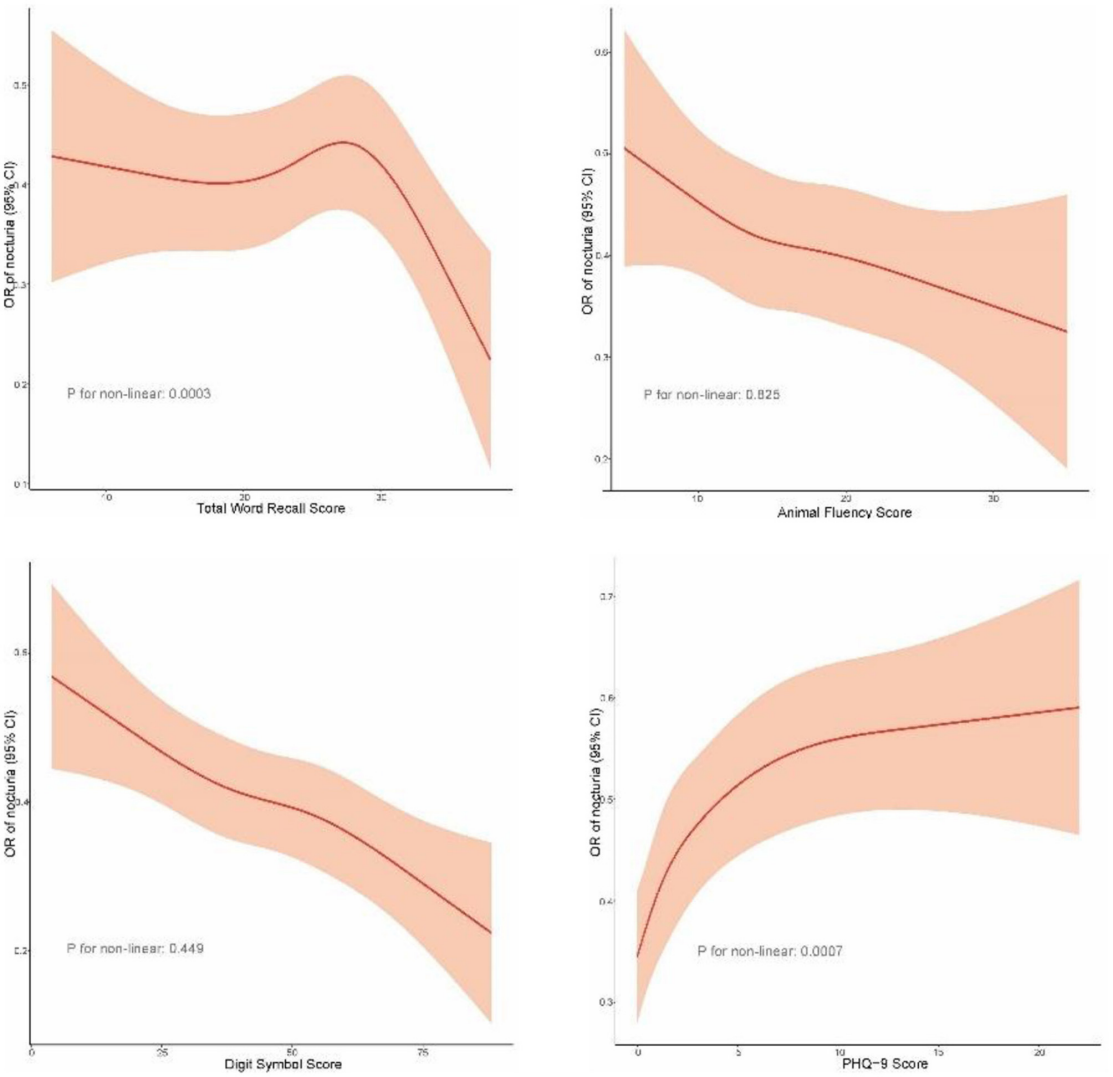
Relationship of cognitive function and depression scores with the risk of nocturia in older adults.

Restricted cubic spline curve showed that TWRS, and PHQ-9 scores had non-linear relationships with the risk of nocturia (*p* for non-linear < 0.05). AFS and DSS were linearly associated with nocturia risk (*p* for non-linear > 0.05). And the risk of nocturia decreased by ~1% for every one-point increase in DSS (Figure 2). There was no significant association between AFS and nocturia risk.

The association of TWRS, AFS, DSS, and PHQ-9 scores with nocturia risk stratified by male/female was shown in Table 3. The results showed that the associations were significant in the unadjusted model. In the adjusted models, PHQ-9 scores were positively significantly associated with the risk of nocturia in both males and females. There was a significant correlation between AFS, DSS, and the risk of nocturia in females (*p* < 0.05); specifically, lower scores were associated with a higher risk of nocturia. In contrast, no significant correlation was observed in males.

**Table 3:** Association of cognitive function and depression scores with nocturia risk stratified by male/female.

**Cognitive function and depression scores**	**Model**	**Males**		**Females**	
		**Odds ratio (95% confidence interval)**	***p*** **-Value**	**Odds ratio (95% confidence interval)**	***p*** **-Value**
Total word recall score	Unadjusted	0.9711 (0.9546, 0.9879)	**0.0008**	0.9655 (0.9505, 0.9808)	**<0.0001**
	Model I	0.9973 (0.9775, 1.0174)	0.7896	0.9859 (0.9678, 1.0045)	0.1359
	Model II	0.9950 (0.9744, 1.0159)	0.6352	0.9869 (0.9678, 1.0064)	0.1873
Animal fluency score	Unadjusted	0.9603 (0.9418, 0.9792)	**<0.0001**	0.9329 (0.9143, 0.9519)	**<0.000001**
	Model I	0.9946 (0.9721, 1.0176)	0.6398	0.9686 (0.9458, 0.9921)	**0.0090**
	Model II	0.9952 (0.9717, 1.0193)	0.6929	0.9724 (0.9480, 0.9973)	**0.0302**
Digit symbol score	Unadjusted	0.9766 (0.9700, 0.9833)	**<0.000001**	0.9735 (0.9676, 0.9794)	**<0.000001**
	Model I	0.9893 (0.9797, 0.9989)	**0.0294**	0.9851 (0.9768, 0.9935)	**0.0005**
	Model II	0.9907 (0.9805, 1.0009)	0.0745	0.9863 (0.9773, 0.9955)	**0.0034**
9-item patient health questionnaire score	Unadjusted	1.1047 (1.0728, 1.1375)	**<0.000001**	1.0809 (1.0556, 1.1067)	**<0.000001**
	Model I	1.1109 (1.0759, 1.1471)	**<0.000001**	1.0606 (1.0328, 1.0890)	**<0.0001**
	Model II	1.0971 (1.0617, 1.1336)	**<0.000001**	1.0578 (1.0286, 1.0879)	**<0.0001**

Model I was adjusted for male/female, age, race, education level, marital status, and poverty-to-income ratio.

Model II was adjusted for male/female, age, race, education level, marital status, and poverty-to-income ratio, body mass index, hypertension, diabetes, smoking status, alcohol drinking status, work activities, and recreational activities.

The bold values were considered statistically significant.

Subgroup analysis of age showed that the adjusted TWRS, DSS and PHQ-9 Score were significantly correlated with the risk of nocturia in the 60–69 years group (*p* < 0.05). Only the PHQ-9 Score was significantly correlated with the risk of nocturia in the 70–80 years group (*p* < 0.05, Table 4).

**Table 4:** Association of cognitive function and depression scores with nocturia risk stratified by age.

**Cognitive function and depression scores**	**Model**	**Age (60–69 years)**		**Age (70–80 years)**	
		**OR (95% CI)**	***p*** **-Value**	**OR (95% CI)**	***p*** **-Value**
Total word recall score	Non-adjusted	0.9588 (0.9431, 0.9748)	**<0.000001**	0.9862 (0.9700, 1.0025)	0.09746
	Adjust I	0.9785 (0.9596, 0.9978)	**0.029319**	0.9983 (0.9800, 1.0168)	0.85313
	Adjust II	0.9787 (0.9589, 0.9989)	**0.039188**	1.0007 (0.9812, 1.0205)	0.94732
Animal fluency score	Non-adjusted	0.9405 (0.9229, 0.9584)	**<0.000001**	0.9631 (0.9425, 0.9842)	**0.000668**
	Adjust I	0.9796 (0.9581, 1.0017)	0.069585	0.9834 (0.9598, 1.0076)	0.177499
	Adjust II	0.9845 (0.9618, 1.0077)	0.189148	0.9876 (0.9621, 1.0137)	0.347625
Digit symbol score	Non-adjusted	0.9727 (0.9668, 0.9787)	**<0.000001**	0.9804 (0.9735, 0.9873)	**<0.000001**
	Adjust I	0.9864 (0.9779, 0.9949)	**0.001854**	0.9877 (0.9789, 0.9966)	**0.006738**
	Adjust II	0.9879 (0.9788, 0.9970)	**0.009101**	0.9908 (0.9811, 1.0007)	0.069238
PHQ-9 score	Non-adjusted	1.1044 (1.0790, 1.1304)	**<0.000001**	1.0713 (1.0404, 1.1032)	**0.000004**
	Adjust I	1.0874 (1.0593, 1.1161)	**<0.000001**	1.0726 (1.0388, 1.1076)	**0.000019**
	Adjust II	1.0775 (1.0486, 1.1073)	**<0.000001**	1.0678 (1.0324, 1.1044)	**0.000139**

Model I was adjusted for male/female, age, race, education level, marital status, and poverty-to-income ratio.

Model II was adjusted for male/female, age, race, education level, marital status, and poverty-to-income ratio, body mass index, hypertension, diabetes, smoking status, alcohol drinking status, work activities, and recreational activities.

The bold values were considered statistically significant.

**Figure 3:**
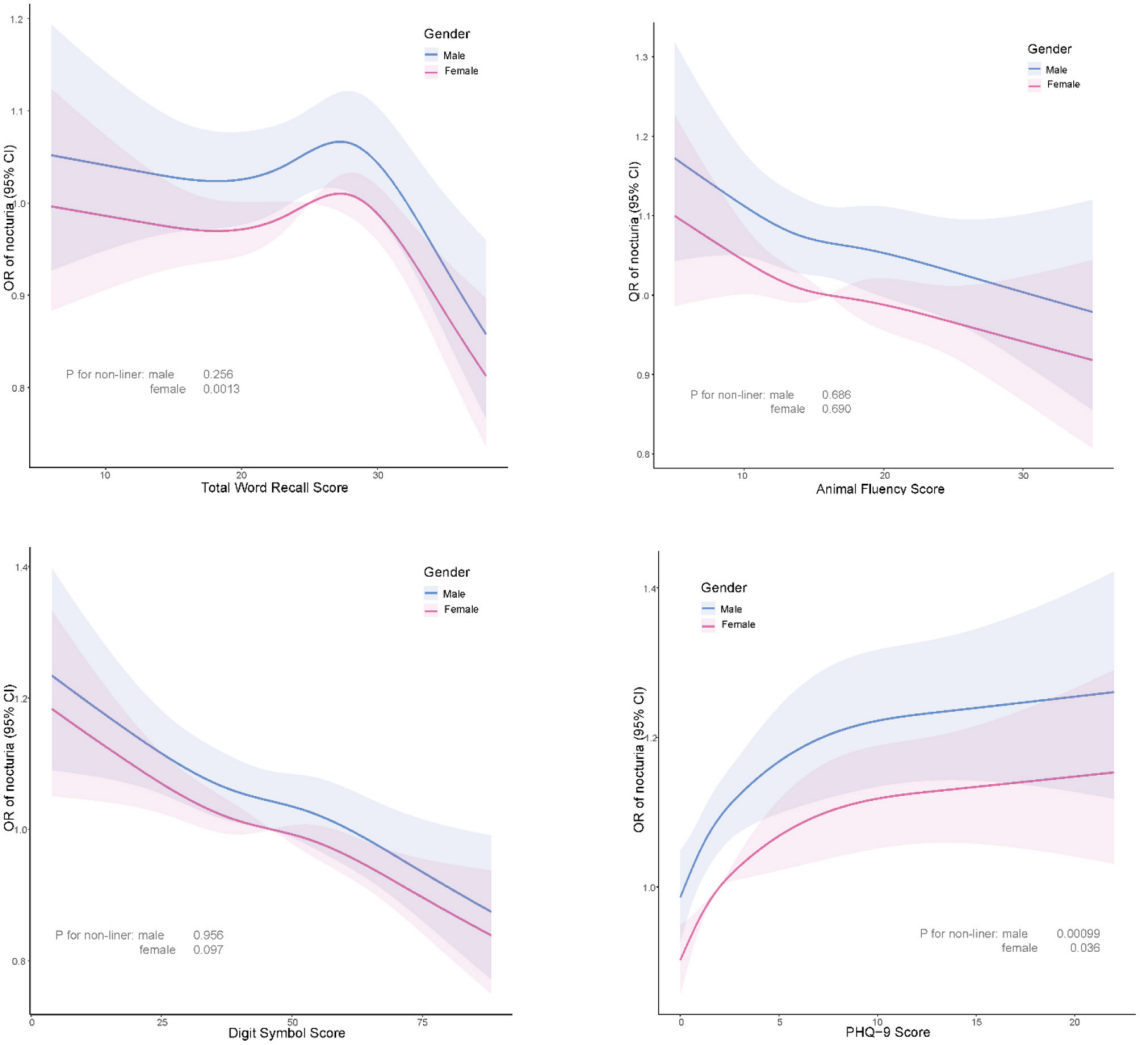
Linear relationship of cognitive function and depression scores with nocturia risk in older adults of both males and females.

The relationships of cognitive function (TWRS, AFS, and DSS) with nocturia risk were linear in males (*p* for non-linear > 0.05). In females, TWRS, and PHQ-9 scores were non-linearly correlated with the risk of nocturia, while DSS and AFS were linearly associated with the risk of nocturia (Figure 3). For every 1-point increase in DSS and AFS in women, the risk of nocturia was reduced by ~2.8 and 1.4%, respectively (*p* < 0.05). TWRS according to threshold values affected the risk of nocturia in females. TWRS >30 decreased the risk of nocturia by ~14% (Table 5). PHQ-9 scores at thresholds of 2 for men and 1 for women indicate a significant association with nocturia. Specifically, when PHQ-9 scores are below these thresholds (i.e., below two for men and below one for women), the risk of nocturia increases by 38% in men and 48% in women, respectively. Conversely, when PHQ-9 scores exceed the respective thresholds, the risk of nocturia increases by 5% in men and 4% in women (Tables 5, 6).

**Table 5:** Association of cognitive function scores/PHQ-9 score with nocturia risk according to threshold values in females.

**Variable**	**Odds ratio (95% confidence interval)**	***p*-Value**
**Total word recall score**		
< 30	1.0162 (0.9911, 1.0419)	0.2073
>30	0.8602 (0.7964, 0.9291)	**0.0001**
**PHQ-9 score**		
<1	1.4786 (1.1003, 1.9869)	**0.0095**
>1	1.0388 (1.0066, 1.0720)	**0.0178**

The bold values were considered statistically significant.

**Table 6:** Association of PHQ-9 score with nocturia risk according to threshold values in males.

**Variable**	**Odds ratio (95% confidence interval)**	***p*-Value**
**PHQ-9 score**		
<2	1.3854 (1.1901, 1.6126)	**<0.0001**
>2	1.0507 (1.0087, 1.0945)	**0.0175**

The bold values were considered statistically significant.

**Figure 4:**
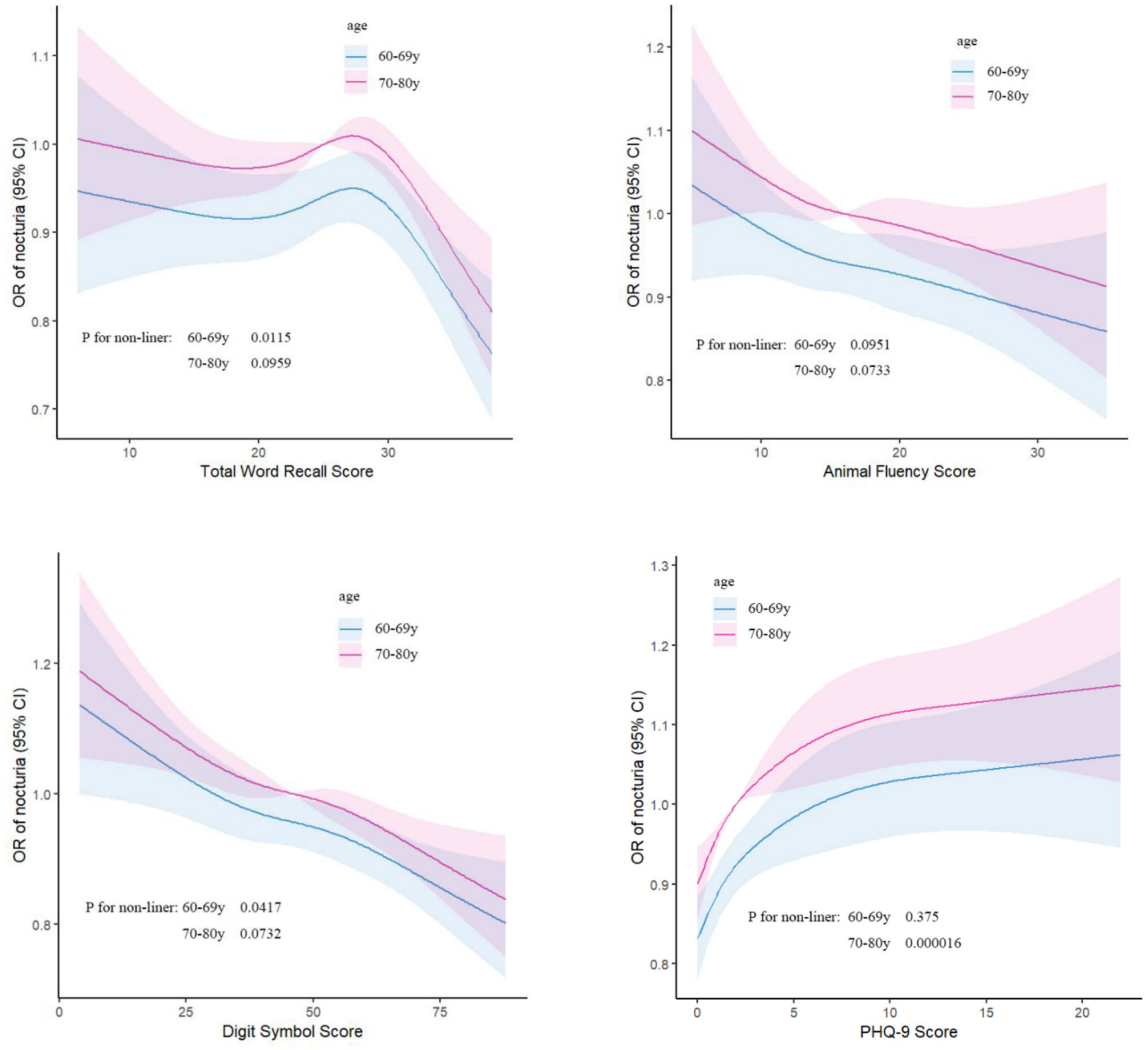
Linear relationship of cognitive function and depression scores with nocturia risk in older adults of different age groups.

There was a linear relationship between AFS and nocturia risk in different age groups (Figure 4). TWRS, and DSS were non-linear correlated with the risk of nocturia in 60–69 years group. When TWRS and DSS were >34 and 67, the risk of nocturia was increased by 33 and 4%, respectively (Table 7). The PHQ-9 Score in the 70–80 years group showed a non-linear relationship with the risk of nocturia. When the PHQ-9 Score was < 1, the risk of nocturia increased by 109% (Table 8).

**Table 7:** Association of cognitive function scores with nocturia risk according to threshold values in 60–69 years.

**Variable**	**Odds ratio (95% confidence interval)**	***p*-Value**
**Total word recall score**		
<34	0.9927 (0.9709, 1.0151)	0.522
>34	0.6675 (0.5163, 0.8630)	**0.002**
**Digit symbol score**		
<67	0.9919 (0.9814, 1.0025)	0.132
>67	0.9612 (0.9251, 0.9988)	**0.0431**

The bold values were considered statistically significant.

**Table 8:** Association of PHQ-9 score with nocturia risk according to threshold values in 70–80 years.

**Variable**	**Odds ratio (95% confidence interval)**	***p*-Value**
**PHQ-9 score**		
<1	2.0936 (1.5695, 2.7927)	**<0.0001**
>1	1.0193 (0.9839, 1.0559)	0.2898

The bold values were considered statistically significant.

### 3.3 Analysis of mediating effects

We assessed the mediating effects of nutritional indexes on the relationship between nocturia, depression, and cognitive function. A bias-corrected bootstrap analysis at a 95% confidence interval based on 5,000 bootstrapped resamples was performed using PROCESS version 4.0, which did not rely on the assumption of normal distribution (31).

The previous results showed the signification correlation between nutritional indices (ALB, HGB) and nocturia (OR = −0.34, SE = 0.133, *p* = 0.01; OR = −0.085, SE = 0.032, *p* < 0.01). To further explore the mechanisms between cognitive function, depression and nocturia risk, we analyzed the mediating effects of ALB and HGB on these correlations.

**Figure 5:**
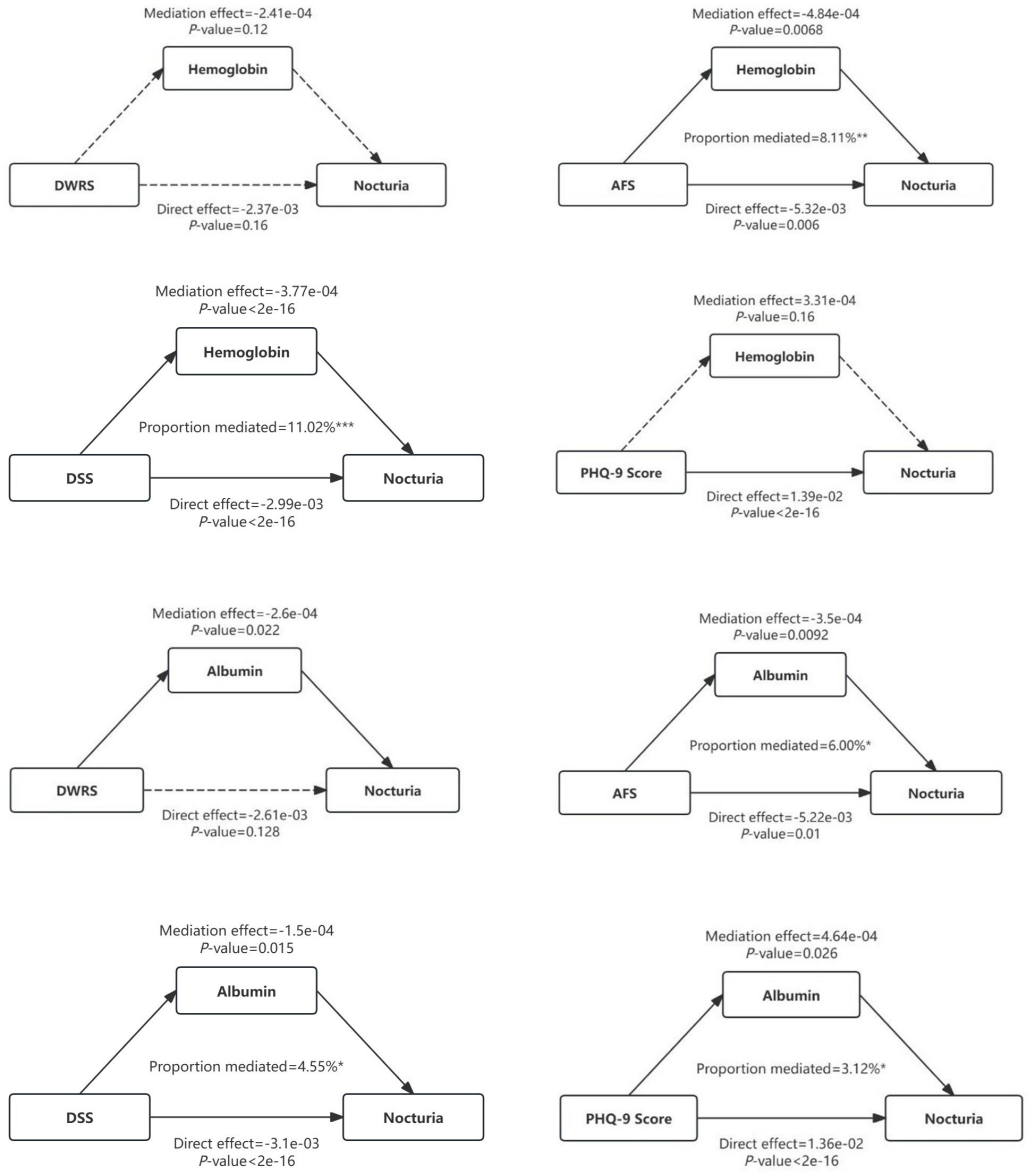
Mediating effects of hemoglobin and albumin on the relationship of cognitive function and depression scores with the risk of nocturia in older adults (*p < 0.05; **p < 0.01; ***p < 0.001).

Figure 5 showed that the direct effect of AFS on the risk of nocturia was −0.005327 (*p* = 0.006), the mediating effect of HGB was −0.000484 (*p* = 0.0068) and the mediating effect accounted for 8.11% (*p* = 0.0088). Meanwhile, HGB had a partial mediating effect on the correlation of DSS with nocturia risk, accounting for 11.02%. However, the mediating effect of HGB on the correlation of PHQ-9, DWRS with nocturia was not significant.

Similarly, ALB had a partial mediating effect on the correlation of AFS, DSS and PHQ-9 scores with nocturia risk in participants, accounting for 1.52, 1.50, and 2.6%, respectively. However, ALB did not mediate the association of DWRS with nocturia risk (Figure 5).

Subgroup analyses of male/female and age were used to study the stability and specificity of mediating effects. Subgroup analysis of male/female showed that HGB only played a partial mediating role in the correlation between AFS and nocturia in women, accounting for 6.60% (*p* = 0.026). Meanwhile, HGB only played a partial mediating role in the correlation between male PHQ-9 Score and nocturia, accounting for 4.07% (*p* = 0.02). HGB played a partial mediating role in the correlation between male and female DSS and nocturia, accounting for 13.4 and 6.81%, respectively (Table 5). ALB only partially mediated the correlation between AFS, DSS, PHQ-9 Score and nocturia risk in women, accounting for 5.00, 4.39 and 4.62%, respectively (Tables 9–12).

**Table 9:** Mediating effects of hemoglobin on the relationship of cognitive function and depression scores with the risk of nocturia in males.

**Mediated**	**Exposure**	**Estimate**	**95% CI lower**	**95% CI upper**	***p*-Value**
Hemoglobin	DWRS				
	ACME (control)	−0.000307	−0.000882	0	0.17
	ACME (treated)	−0.000308	−0.000882	0	0.17
	ADE (control)	−0.00122	−0.005905	0	0.62
	ADE (treated)	−0.00122	−0.005907	0	0.62
	Total effect	−0.001527	−0.006308	0	0.54
	Prop. mediated (control)	0.071636	−1.455422	1.95	0.6
	Prop. mediated (treated)	0.071845	−1.455558	1.95	0.6
	ACME (average)	−0.000308	−0.000882	0	0.17
	ADE (average)	−0.00122	−0.005906	0	0.62
	Prop. mediated (average)	0.07174	−1.45549	1.95	0.6
	AFS				
	ACME (control)	−0.000197	−0.000779	0	0.41
	ACME (treated)	−0.000197	−0.00078	0	0.41
	ADE (control)	−0.002546	−0.007949	0	0.38
	ADE (treated)	−0.002546	−0.007951	0	0.38
	Total effect	−0.002743	−0.008235	0	0.34
	Prop. mediated (control)	0.041829	−0.759598	1	0.58
	Prop. mediated (treated)	0.041894	−0.760016	1	0.58
	ACME (average)	−0.000197	−0.00078	0	0.41
	ADE (average)	−0.002546	−0.00795	0	0.38
	Prop. mediated (average)	0.041861	−0.759807	1	0.58
	DSS				
	ACME (control)	−0.00043	−0.000832	0	**0.0068**
	ACME (treated)	−0.000431	−0.000833	0	**0.0068**
	ADE (control)	−0.002661	−0.004536	0	**0.0172**
	ADE (treated)	−0.002662	−0.004538	0	**0.0172**
	Total effect	−0.003092	−0.004907	0	**0.0048**
	Prop. mediated (control)	0.134157	0.031391	0.49	**0.0116**
	Prop. mediated (treated)	0.13438	0.031504	0.49	**0.0116**
	ACME (average)	−0.000431	−0.000833	0	**0.0068**
	ADE (average)	−0.002662	−0.004536	0	**0.0172**
	Prop. mediated (average)	0.134268	0.031438	0.49	**0.0116**
	PHQ-9 score				
	ACME (control)	0.00079	0.0000932	0	**0.02**
	ACME (treated)	0.000797	0.0000938	0	**0.02**
	ADE (control)	0.0177	0.00904	0.03	**<2e−16**
	ADE (treated)	0.0177	0.00904	0.03	**<2e−16**
	Total effect	0.0185	0.00991	0.03	**<2e−16**
	Prop. mediated (control)	0.0405	0.00503	0.12	**0.02**
	Prop. mediated (treated)	0.0408	0.00508	0.12	**0.02**
	ACME (average)	0.000793	0.0000935	0	**0.02**
	ADE (average)	0.0177	0.00904	0.03	**<2e−16**
	Prop. mediated (average)	0.0407	0.00506	0.12	**0.02**

The bold values were considered statistically significant.

**Table 10:** Mediating effects of hemoglobin on the relationship of cognitive function and depression scores with the risk of nocturia in females.

**Mediated**	**Exposure**	**Estimate**	**95% CI lower**	**95% CI upper**	***p*-Value**
Hemoglobin	DWRS				
	ACME (control)	−0.00014	−0.000569	0	0.41
	ACME (treated)	−0.00014	−0.00057	0	0.41
	ADE (control)	−0.00329	−0.007351	0	0.15
	ADE (treated)	−0.00329	−0.007352	0	0.15
	Total effect	−0.00343	−0.007494	0	0.13
	Prop. mediated (control)	0.030897	−0.26367	0.42	0.47
	Prop. mediated (treated)	0.031042	−0.263724	0.42	0.47
	ACME (average)	−0.00014	−0.000569	0	0.41
	ADE (average)	−0.00329	−0.007352	0	0.15
	Prop. mediated (average)	0.03097	−0.263697	0.42	0.47
	AFS				
	ACME (control)	−0.000608	−0.00135	0	**0.024**
	ACME (treated)	−0.000612	−0.001358	0	**0.024**
	ADE (control)	−0.00817	−0.01294	0	**0.0044**
	ADE (treated)	−0.008173	−0.012948	0	**0.0044**
	Total effect	−0.008782	−0.013552	0	**0.002**
	Prop. mediated (control)	0.065739	0.006971	0.25	**0.026**
	Prop. mediated (treated)	0.066224	0.007078	0.25	**0.026**
	ACME (average)	−0.00061	−0.001356	0	**0.024**
	ADE (average)	−0.008171	−0.012945	0	**0.0044**
	Prop. mediated (average)	0.065981	0.007025	0.25	**0.026**
	DSS				
	ACME (control)	−0.000263	−0.000564	0	**0.0364**
	ACME (treated)	−0.000264	−0.000565	0	**0.0364**
	ADE (control)	−0.00348	−0.004887	0	**0.0016**
	ADE (treated)	−0.003481	−0.004888	0	**0.0016**
	Total effect	−0.003744	−0.005123	0	**<2e−16**
	Prop. mediated (control)	0.068026	0.003502	0.19	**0.0364**
	Prop. mediated (treated)	0.06824	0.003522	0.19	**0.0364**
	ACME (average)	−0.000264	−0.000564	0	**0.0364**
	ADE (average)	−0.003481	−0.004888	0	**0.0016**
	Prop. mediated (average)	0.068133	0.003512	0.19	**0.0364**
	PHQ-9 score				
	ACME (control)	−3.63E-05	−5.97E-04	0	0.876
	ACME (treated)	−3.66E-05	−6.01E-04	0	0.876
	ADE (control)	1.18E-02	2.23E-03	0.02	**0.016**
	ADE (treated)	1.18E-02	2.23E-03	0.02	**0.016**
	Total effect	1.17E-02	2.16E-03	0.02	**0.015**
	Prop. mediated (control)	−2.71E-03	−8.31E-02	0.07	0.88
	Prop. mediated (treated)	−2.75E-03	−8.32E-02	0.07	0.88
	ACME (average)	−3.65E-05	−5.99E-04	0	0.876
	ADE (average)	1.18E-02	2.23E-03	0.02	**0.016**
	Prop. mediated (average)	−2.73E-03	−8.32E-02	0.07	0.88

The bold values were considered statistically significant.

**Table 11:** Mediating effects of albumin on the relationship of cognitive function and depression scores with the risk of nocturia in males.

**Mediated**	**Exposure**	**Estimate**	**95% CI lower**	**95% CI upper**	***p*-Value**
Albumin	DWRS				
	ACME (control)	−0.00019	−0.000655	0	0.26
	ACME (treated)	−0.00019	−0.000655	0	0.26
	ADE (control)	−0.001562	−0.006318	0	0.55
	ADE (treated)	−0.001562	−0.006319	0	0.55
	Total effect	−0.001752	−0.006464	0	0.5
	Prop. mediated (control)	0.036356	−0.886118	1.08	0.61
	Prop. mediated (treated)	0.036449	−0.886158	1.08	0.61
	ACME (average)	−0.00019	−0.000655	0	0.26
	ADE (average)	−0.001562	−0.006319	0	0.55
	Prop. mediated (average)	0.036402	−0.886138	1.08	0.61
	AFS				
	ACME (control)	−0.000275	−0.000804	0	0.13
	ACME (treated)	−0.000275	−0.000805	0	0.13
	ADE (control)	−0.002345	−0.007756	0	0.4
	ADE (treated)	−0.002345	−0.007755	0	0.4
	Total effect	−0.00262	−0.007981	0	0.34
	Prop. mediated (control)	0.056633	−1.033905	1.21	0.43
	Prop. mediated (treated)	0.056788	−1.033961	1.21	0.43
	ACME (average)	−0.000275	−0.000805	0	0.13
	ADE (average)	−0.002345	−0.007755	0	0.4
	Prop. mediated (average)	0.056711	−1.033933	1.21	0.43
	DSS				
	ACME (control)	−0.00012	−0.000373	0	0.164
	ACME (treated)	−0.00012	−0.000373	0	0.164
	ADE (control)	−0.002628	−0.004587	0	**0.027**
	ADE (treated)	−0.002628	−0.004586	0	**0.027**
	Total effect	−0.002748	−0.004688	0	**0.02**
	Prop. mediated (control)	0.038202	−0.026364	0.23	0.181
	Prop. mediated (treated)	0.038239	−0.026394	0.23	0.181
	ACME (average)	−0.00012	−0.000373	0	0.164
	ADE (average)	−0.002628	−0.004586	0	**0.027**
	Prop. mediated (average)	0.038221	−0.026379	0.23	0.181
	PHQ-9 score				
	ACME (control)	0.00038	−0.000338	0	0.3
	ACME (treated)	0.000384	−0.000341	0	0.3
	ADE (control)	0.01864	0.00989	0.03	**<2e−16**
	ADE (treated)	0.018644	0.009891	0.03	**<2e−16**
	Total effect	0.019024	0.010283	0.03	**<2e−16**
	Prop. mediated (control)	0.017197	−0.019335	0.08	0.3
	Prop. mediated (treated)	0.017378	−0.019467	0.08	0.3
	ACME (average)	0.000382	−0.000339	0	0.3
	ADE (average)	0.018642	0.009891	0.03	**<2e−16**
	Prop. mediated (average)	0.017287	−0.019401	0.08	0.3

The bold values were considered statistically significant.

**Table 12:** Mediating effects of albumin on the relationship of cognitive function and depression scores with the risk of nocturia in females.

**Mediated**	**Exposure**	**Estimate**	**95% CI lower**	**95% CI upper**	***p*-Value**
Albumin	DWRS				
	ACME (control)	−0.000391	−0.00096	0	**0.028**
	ACME (treated)	−0.000392	−0.000962	0	**0.028**
	ADE (control)	−0.003383	−0.007476	0	0.144
	ADE (treated)	−0.003384	−0.007479	0	0.144
	Total effect	−0.003774	−0.007888	0	0.105
	Prop. mediated (control)	0.087735	−0.424741	0.82	0.128
	Prop. mediated (treated)	0.087931	−0.425025	0.82	0.128
	ACME (average)	−0.000391	−0.000962	0	**0.028**
	ADE (average)	−0.003383	−0.007478	0	0.144
	Prop. mediated (average)	0.087833	−0.424883	0.82	0.128
	AFS				
	ACME (control)	−0.000455	−0.001074	0	**0.0384**
	ACME (treated)	−0.000457	−0.001079	0	**0.0384**
	ADE (control)	−0.007997	−0.012923	0	**0.0056**
	ADE (treated)	−0.008	−0.012927	0	**0.0056**
	Total effect	−0.008454	−0.013366	0	**0.0024**
	Prop. mediated (control)	0.049899	0.002235	0.2	**0.0408**
	Prop. mediated (treated)	0.050115	0.002245	0.2	**0.0408**
	ACME (average)	−0.000456	−0.001077	0	**0.0384**
	ADE (average)	−0.007998	−0.012924	0	**0.0056**
	Prop. mediated (average)	0.050007	0.00224	0.2	**0.0408**
	DSS				
	ACME (control)	−0.000176	−0.000403	0	**0.0268**
	ACME (treated)	−0.000176	−0.000405	0	**0.0268**
	ADE (control)	−0.003615	−0.005015	0	**0.0004**
	ADE (treated)	−0.003615	−0.005017	0	**0.0004**
	Total effect	−0.003791	−0.005175	0	**<2e−16**
	Prop. mediated (control)	0.043821	0.002878	0.13	**0.0268**
	Prop. mediated (treated)	0.044021	0.002888	0.13	**0.0268**
	ACME (average)	−0.000176	−0.000405	0	**0.0268**
	ADE (average)	−0.003615	−0.005016	0	**0.0004**
	Prop. mediated (average)	0.043921	0.002883	0.13	**0.0268**
	PHQ-9 score				
	ACME (control)	5.59E-04	3.99E-05	0	**0.028**
	ACME (treated)	5.63E-04	4.06E-05	0	**0.028**
	ADE (control)	1.07E-02	1.23E-03	0.02	**0.031**
	ADE (treated)	1.07E-02	1.23E-03	0.02	**0.031**
	Total effect	1.13E-02	1.89E-03	0.02	**0.024**
	Prop. mediated (control)	4.60E-02	−5.07E-04	0.24	0.052
	Prop. mediated (treated)	4.64E-02	−5.08E-04	0.24	0.052
	ACME (average)	5.61E-04	4.02E-05	0	**0.028**
	ADE (average)	1.07E-02	1.23E-03	0.02	**0.031**
	Prop. mediated (average)	4.62E-02	−5.08E-04	0.24	0.052

The bold values were considered statistically significant.

Age subgroup analysis showed that HGB had a direct effect of −0.002283 (*p* = 0.0284) and a mediating effect of −0.000647 (*p* < 2e−16) in the correlation between DSS and nocturia risk in the 70–80 years group, accounting for 21.5% (*p* = 0.0052, Table 7). Similarly, albumin played a partial mediating role in the correlation between DSS and diabetes risk in the 70–80 years group, accounting for 8.07% (*p* = 0.041, Table 8). These results indicate that the correlation of AFS, DSS, and PHQ-9 scores with nocturia risk may be mediated by nutritional status (Tables 13–16).

**Table 13:** Mediating effects of hemoglobin on the relationship of cognitive function and depression scores with the risk of nocturia in 60–69 years.

**Mediated**	**Exposure**	**Estimate**	**95% CI lower**	**95% CI upper**	***p*-Value**
Hemoglobin	DWRS				
	ACME (control)	−0.00026	−0.000728	0	0.096
	ACME (treated)	−0.00026	−0.00073	0	0.096
	ADE (control)	−0.00476	−0.00868	0	**0.036**
	ADE (treated)	−0.004761	−0.008681	0	**0.036**
	Total effect	−0.00502	−0.00896	0	**0.028**
	Prop. mediated (control)	0.045988	−0.017433	0.28	0.12
	Prop. mediated (treated)	0.046083	−0.017432	0.28	0.12
	ACME (average)	−0.00026	−0.000729	0	0.096
	ADE (average)	−0.004761	−0.008681	0	**0.036**
	Prop. mediated (average)	0.046036	−0.017432	0.28	0.12
	AFS				
	ACME (control)	−0.000303	−0.000808	0	0.093
	ACME (treated)	−0.000304	−0.000809	0	0.093
	ADE (control)	−0.005519	−0.010449	0	**0.034**
	ADE (treated)	−0.005519	−0.01045	0	**0.034**
	Total effect	−0.005823	−0.010784	0	**0.027**
	Prop. mediated (control)	0.046852	−0.014976	0.28	0.118
	Prop. mediated (treated)	0.046883	−0.014993	0.28	0.118
	ACME (average)	−0.000303	−0.000809	0	0.093
	ADE (average)	−0.005519	−0.01045	0	**0.034**
	Prop. mediated (average)	0.046867	−0.014984	0.28	0.118
	DSS				
	ACME (control)	−0.000203	−0.000519	0	0.1532
	ACME (treated)	−0.000203	−0.00052	0	0.1532
	ADE (control)	−0.00324	−0.004886	0	**0.0008**
	ADE (treated)	−0.003241	−0.004886	0	**0.0008**
	Total effect	−0.003444	−0.005039	0	**0.0008**
	Prop. mediated (control)	0.056314	−0.022787	0.21	0.154
	Prop. mediated (treated)	0.056417	−0.022814	0.21	0.154
	ACME (average)	−0.000203	−0.000519	0	0.1532
	ADE (average)	−0.003241	−0.004886	0	**0.0008**
	Prop. mediated (average)	0.056365	−0.0228	0.21	0.154
	PHQ-9 score				
	ACME (control)	0.000244	−0.00011	0	0.22
	ACME (treated)	0.000247	−0.000112	0	0.22
	ADE (control)	0.014503	0.008395	0.02	**<2e−16**
	ADE (treated)	0.014506	0.0084	0.02	**<2e−16**
	Total effect	0.01475	0.008664	0.02	**<2e−16**
	Prop. mediated (control)	0.013558	−0.007588	0.06	0.22
	Prop. mediated (treated)	0.013745	−0.007703	0.06	0.22
	ACME (average)	0.000246	−0.000111	0	0.22
	ADE (average)	0.014504	0.008397	0.02	**<2e−16**
	Prop. mediated (average)	0.013651	−0.007646	0.06	0.22

The bold values were considered statistically significant.

**Table 14:** Mediating effects of hemoglobin on the relationship of cognitive function and depression scores with the risk of nocturia in 70–80 years.

**Mediated**	**Exposure**	**Estimate**	**95% CI lower**	**95% Ci upper**	***p*-Value**
Hemoglobin	DWRS				
	ACME (control)	−0.000199	−0.000851	0	0.52
	ACME (treated)	−0.000199	−0.000852	0	0.52
	ADE (control)	−0.000273	−0.00495	0	0.91
	ADE (treated)	−0.000273	−0.00495	0	0.91
	Total effect	−0.000472	−0.005173	0	0.84
	Prop. mediated (control)	0.027307	−2.046913	1.99	0.87
	Prop. mediated (treated)	0.02738	−2.046584	1.99	0.87
	ACME (average)	−0.000199	−0.000852	0	0.52
	ADE (average)	−0.000273	−0.00495	0	0.91
	Prop. mediated (average)	0.027343	−2.046749	1.99	0.87
	AFS				
	ACME (control)	−0.000794	−0.001768	0	**0.042**
	ACME (treated)	−0.000797	−0.001777	0	**0.042**
	ADE (control)	−0.004317	−0.009891	0	0.156
	ADE (treated)	−0.00432	−0.009897	0	0.156
	Total effect	−0.005114	−0.01058	0	0.097
	Prop. mediated (control)	0.137764	−0.518599	1.17	0.129
	Prop. mediated (treated)	0.138198	−0.518758	1.17	0.129
	ACME (average)	−0.000796	−0.001771	0	**0.042**
	ADE (average)	−0.004319	−0.009894	0	0.156
	Prop. mediated (average)	0.137981	−0.518678	1.17	0.129
	DSS				
	ACME (control)	−0.000646	−0.001092	0	**<2e−16**
	ACME (treated)	−0.000648	−0.001094	0	**<2e−16**
	ADE (control)	−0.002282	−0.004097	0	**0.0284**
	ADE (treated)	−0.002283	−0.0041	0	**0.0284**
	Total effect	−0.002929	−0.004674	0	**0.0052**
	Prop. mediated (control)	0.215117	0.083853	0.72	**0.0052**
	Prop. mediated (treated)	0.215622	0.084378	0.72	**0.0052**
	ACME (average)	−0.000647	−0.001093	0	**<2e−16**
	ADE (average)	−0.002283	−0.004099	0	**0.0284**
	Prop. mediated (average)	0.215369	0.084088	0.72	**0.0052**
	PHQ-9 score				
	ACME (control)	0.000156	−0.000933	0	0.771
	ACME (treated)	0.000156	−0.000935	0	0.771
	ADE (control)	0.012705	−0.000487	0.03	0.058
	ADE (treated)	0.012706	−0.000487	0.03	0.058
	Total effect	0.012861	−0.000356	0.03	0.058
	Prop. mediated (control)	0.010829	−0.181025	0.23	0.773
	Prop. mediated (treated)	0.010854	−0.18098	0.23	0.773
	ACME (average)	0.000156	−0.000934	0	0.771
	ADE (average)	0.012705	−0.000487	0.03	0.058
	Prop. mediated (average)	0.010842	−0.181003	0.23	0.773

The bold values were considered statistically significant.

**Table 15:** Mediating effects of albumin on the relationship of cognitive function and depression scores with the risk of nocturia in 60–69 years.

**Mediated**	**Exposure**	**Estimate**	**95% CI lower**	**95% CI upper**	***p*-Value**
Albumin	DWRS				
	ACME (control)	−0.000216	−0.000673	0	0.171
	ACME (treated)	−0.000216	−0.000675	0	0.171
	ADE (control)	−0.005368	−0.009227	0	**0.018**
	ADE (treated)	−0.005369	−0.009228	0	**0.018**
	Total effect	−0.005585	−0.009448	0	**0.013**
	Prop. mediated (control)	0.032989	−0.017131	0.21	0.18
	Prop. mediated (treated)	0.033111	−0.017189	0.21	0.18
	ACME (average)	−0.000216	−0.000675	0	0.171
	ADE (average)	−0.005369	−0.009228	0	**0.018**
	Prop. mediated (average)	0.03305	−0.01716	0.21	0.18
	AFS				
	ACME (control)	−0.00027	−0.000793	0	0.11
	ACME (treated)	−0.00027	−0.000793	0	0.11
	ADE (control)	−0.005106	−0.010247	0	0.058
	ADE (treated)	−0.005106	−0.010248	0	0.058
	Total effect	−0.005376	−0.010477	0	**0.046**
	Prop. mediated (control)	0.043414	−0.04173	0.38	0.148
	Prop. mediated (treated)	0.043398	−0.041686	0.38	0.148
	ACME (average)	−0.00027	−0.000793	0	0.11
	ADE (average)	−0.005106	−0.010247	0	0.058
	Prop. mediated (average)	0.043406	−0.041708	0.38	0.148
	DSS				
	ACME (control)	−0.000148	−0.000397	0	0.1344
	ACME (treated)	−0.000148	−0.000398	0	0.1344
	ADE (control)	−0.003148	−0.004814	0	**0.0016**
	ADE (treated)	−0.003148	−0.004814	0	**0.0016**
	Total effect	−0.003296	−0.004955	0	**0.0012**
	Prop. mediated (control)	0.042024	−0.013246	0.16	0.1356
	Prop. mediated (treated)	0.042113	−0.013302	0.16	0.1356
	ACME (average)	−0.000148	−0.000398	0	0.1344
	ADE (average)	−0.003148	−0.004814	0	**0.0016**
	Prop. mediated (average)	0.042069	−0.013274	0.16	0.1356
	PHQ-9 score				
	ACME (control)	0.000348	−0.000215	0	0.22
	ACME (treated)	0.000353	−0.000219	0	0.22
	ADE (control)	0.014311	0.00846	0.02	**<2e−16**
	ADE (treated)	0.014316	0.008463	0.02	**<2e−16**
	Total effect	0.014664	0.008748	0.02	**<2e−16**
	Prop. mediated (control)	0.02106	−0.015754	0.08	0.22
	Prop. mediated (treated)	0.02137	−0.015896	0.08	0.22
	ACME (average)	0.000351	−0.000217	0	0.22
	ADE (average)	0.014313	0.008462	0.02	**<2e−16**
	Prop. mediated (average)	0.021215	−0.015814	0.08	0.22

The bold values were considered statistically significant.

**Table 16:** Mediating effects of albumin on the relationship of cognitive function and depression scores with the risk of nocturia in 70–80 years.

**Mediated**	**Exposure**	**Estimate**	**95% CI lower**	**95% CI upper**	***p*-Value**
Albumin	DWRS				
	ACME (control)	−0.000442	−0.001075	0	**0.036**
	ACME (treated)	−0.000442	−0.001075	0	**0.036**
	ADE (control)	0.000201	−0.004343	0	0.947
	ADE (treated)	0.000201	−0.004343	0	0.947
	Total effect	−0.000241	−0.004788	0	0.929
	Prop. mediated (control)	0.044816	−2.736136	2.46	0.931
	Prop. mediated (treated)	0.044965	−2.736068	2.46	0.931
	ACME (average)	−0.000442	−0.001075	0	**0.036**
	ADE (average)	0.000201	−0.004343	0	0.947
	Prop. mediated (average)	0.044891	−2.736102	2.46	0.931
	AFS				
	ACME (control)	−0.000618	−0.001394	0	**0.02**
	ACME (treated)	−0.00062	−0.001399	0	**0.02**
	ADE (control)	−0.004113	−0.009793	0	0.19
	ADE (treated)	−0.004115	−0.009797	0	0.19
	Total effect	−0.004733	−0.010327	0	0.13
	Prop. mediated (control)	0.112048	−0.724116	1.22	0.14
	Prop. mediated (treated)	0.11233	−0.724172	1.22	0.14
	ACME (average)	−0.000619	−0.001397	0	**0.02**
	ADE (average)	−0.004114	−0.009795	0	0.19
	Prop. mediated (average)	0.112189	−0.724144	1.22	0.14
	DSS				
	ACME (control)	−0.000238	−0.000546	0	**0.026**
	ACME (treated)	−0.000238	−0.000547	0	**0.026**
	ADE (control)	−0.002495	−0.004327	0	**0.026**
	ADE (treated)	−0.002496	−0.004328	0	**0.026**
	Total effect	−0.002733	−0.004538	0	**0.015**
	Prop. mediated (control)	0.080632	0.003467	0.38	**0.041**
	Prop. mediated (treated)	0.080786	0.003472	0.38	**0.041**
	ACME (average)	−0.000238	−0.000547	0	**0.026**
	ADE (average)	−0.002495	−0.004328	0	**0.026**
	Prop. mediated (average)	0.080709	0.00347	0.38	**0.041**
	PHQ-9 score				
	ACME (control)	0.000692	0.0000294	0	**0.036**
	ACME (treated)	0.000694	0.0000294	0	**0.036**
	ADE (control)	0.0121	−0.00149	0.03	0.082
	ADE (treated)	0.0121	−0.00149	0.03	0.082
	Total effect	0.0128	−0.000736	0.03	0.064
	Prop. mediated (control)	0.0487	−0.121	0.47	0.1
	Prop. mediated (treated)	0.0489	−0.121	0.47	0.1
	ACME (average)	0.000693	0.0000294	0	**0.036**
	ADE (average)	0.0121	−0.00149	0.03	0.082
	Prop. mediated (average)	0.0488	−0.121	0.47	0.1

The bold values were considered statistically significant.

### 3.4 Sensitivity analysis

Cognitive impairment frequently co-occurs with nocturia. Consequently, the relationship between cognitive function and nocturia in individuals without cognitive impairment was examined to evaluate the sensitivity of the study. The findings of the sensitivity analysis revealed a significant correlation between cognitive function in cognitively unimpaired individuals and the risk of nocturia (Supplementary Table S1), aligning with the aforementioned results.

## 4 Discussion

Nocturia was a significant public health problem worldwide. Nocturia was associated with increased all-cause mortality, poor sleep, depression, increased risk of falls and fractures, and reduced quality of life, and the multifactorial nature of the condition complicated its diagnosis and treatment (32). Lower urinary tract symptoms, benign prostatic hyperplasia, hypertension, diabetes, and sleep apnea are risk factors for nocturia (33–35). Although age, comorbidities, and sleep disturbances are widely recognized as common risk factors for nocturia and cognitive dysfunction, research exploring the relationship between nocturia and cognitive dysfunction remains relatively limited, particularly when compared to studies on other lower urinary tract diseases and symptoms (4, 36, 37). A meta-analysis showed that the prevalence of nocturia in older adults with cognitive impairment ranged from 45.8 to 61% (4). The regulation of urethral sphincter, urethra and bladder by neural network controls urine storage and urination (38–41). Among them, the pontine micturition center (PMC) mediated spino-bubo-spinal reflex was the basis of active urination control, and periaqueductal gray (PAG) was the switch to induce urination (42). The function of urination was regulated by PMC, PAG and prefrontal lobe coordination (41–43). The forebrain mediated attention, working memory and executive function through acetylcholine (44). These findings suggested that depression and CI were associated with an increased risk of nocturia. In addition, we found different threshold effects between cognitive function/depression scores and nocturia risk, and keeping scores below the threshold could reduce the incidence of nocturia. Despite the absence of male/female differences in disease prevalence, there were male/female differences in the association of depression and cognitive function on nocturia risk. For instance, AFS, DSS, and PHQ-9 scores were significantly correlated with nocturia risk in women, whereas only PHQ-9 scores were significantly associated with nocturia in men. However, the mechanisms by which this occurs are unclear.

Depression was also a major public health problem worldwide (45). Nocturia was significantly associated with sleep disturbances in a bidirectional manner. Specifically, baseline nocturia was linked to new-onset sleep disturbances (OR = 1.49), while poor baseline sleep quality was associated with the development of new-onset nocturia (OR = 1.26). This effect was more pronounced among women and individuals under the age of 50 (46). Furthermore, sleep disruption may serve as an important mediator in the development of depression (47, 48). In our cohort, depression was significantly associated with nocturia in older adults, consistent with findings in young adults (49). Moreover, there were no male/female differences in the association between depression and nocturia risk. Little is known about the association between cognitive function and nocturia risk. We examined the association of TWRS, AFS, and DSS with nocturia risk and found that AFS, and DSS were associated with the risk of nocturia in women but not in men.

Nutritional status is linked with nocturia, depression, and cognitive function (50–52). We investigated the mediating effect of ALB and HGB on the association between nocturia, depression, and cognitive function. The results suggest that nutritional status partially mediates the relationship between nocturia risk, depression, and cognitive function in women and the association between nocturia risk and depression in men.

Our results showed a strong association between albumin and nocturia and partially mediate the association between cognitive function/depression and nocturia. Chuang et al. (53) found in their study on the correlation between nocturia and cirrhosis that the average albumin level of patients with nocturia and urinary incontinence was 3.85 ± 0.63 g/dl, which was lower than that of the general population. Low albumin levels lead to decreased osmotic pressure, eliminating compensatory diuretic effects, and increased urine excretion (54). On the other hand, there was a significant association between albumin and cholinesterase, which affected bladder function (55, 56).

Hemoglobin concentration was a major factor in determining the delivery of oxygen to tissues (57). While hypoxia may lead to an increase in hemoglobin compensatory (58), malnutrition often resulted in insufficient hemoglobin synthesis raw materials, leaving tissues in a hypoxic state. Andersson et al. (59) found that bladder ischemia and hypoxia may be an important factor affecting bladder function. Therefore, improving anemia and oxygenation by increasing hemoglobin raw material intake may improve the incidence and symptoms of nocturia to some extent.

Currently, research had demonstrated that the mechanism of nocturia in the elderly varies between male/females. In women, the risk of nocturia was linked to a diminished circadian urination rhythm. Specifically, nocturia in older women was associated with reduced estrogen levels and impaired pelvic floor function (60). Notably, the risk of nocturia decreased by 25% (OR = 0.75) in the tertile group with the highest Free Androgen Index (FAI) (61, 62). In men, nocturia was significantly associated with testosterone deficiency (TD), with an odds ratio (OR) of 2.45 for TD and nocturia in patients over 60 years old with cardiovascular disease (CVD) (63, 64). Furthermore, differences exist in the mechanisms underlying bladder dysfunction. Older women were more likely to experience reduced bladder capacity (dBC, OR = 3.00), whereas men exhibit nocturnal bladder overactivity (dNBC, OR = 10.12) (65). Our results suggested that malnutrition also played a role in the onset of nocturia. And there were significant male/female differences. Older women typically exhibited a significantly higher risk of malnutrition compared to their male counterparts. A study conducted in the Basque Country of Spain revealed that the prevalence of malnutrition among women was 16.4% (data for men were inconclusive), with up to 47.5% being at risk (66). Likewise, within the community-dwelling older population, women are eight times more likely to be at risk of malnutrition than men (OR = 8) (67). But in rural Ethiopia, malnutrition rates of 34% for men over 65 and 25% for women show the opposite trend (68).

The study has limitations. First, the experimental design was retrospective. Therefore, longitudinal studies are necessary to confirm our findings. Second, the study did not assess the causal relationship between nocturia, depression, and cognitive function. Third, the effects of other nutritional indicators were not evaluated.

In conclusion, the results suggest that depression and CI are positively significantly associated with the risk of nocturia, especially in women. ALB and HGB levels partially mediate the association between nocturia, depression, and CI in older adults. Thus, improving nutrition may reduce the risk of nocturia, depression, and CI.

## Data Availability

The datasets presented in this study can be found in online repositories. The names of the repository/repositories and accession number(s) can be found below: National Health and Nutrition Examination Survey.
