# Supplementary material for: Relationship between nocturia, depression, and cognitive function and the mediating effects of nutritional indexes in older adults: data from NHANES 2011–2014
Source: Front Nutr. 2025 Jul 2;12:1533683. doi: 10.3389/fnut.2025.1533683 (PMC12263384; doi:10.3389/fnut.2025.1533683)
Supplement: Supplementary file 1 [file Table_1.docx]

**Supplement Table 1.** Demographic characteristics of the non-cognitive impairment.

|  | **No nocturia** | **Nocturia** | **P value** |
| --- | --- | --- | --- |
| ***N*** | 822 | 537 |  |
| **Age [years, medium (IQR)]** | 66.00 (62.00-71.00) | 67.00 (63.00-73.00) | <0.001 |
| **Gender (%)** |  |  | 0.904 |
| Male | 357 (43.43%) | 235 (43.76%) |  |
| Female | 465 (56.57%) | 302 (56.24%) |  |
| **Race/Ethnicity (%)** |  |  | <0.001 |
| Mexican American | 50 (6.08%) | 45 (8.38%) |  |
| Other Hispanic | 53 (6.45%) | 40 (7.45%) |  |
| Non-Hispanic White | 513 (62.41%) | 275 (51.21%) |  |
| Non-Hispanic Black | 128 (15.57%) | 125 (23.28%) |  |
| Other Race | 78 (9.49%) | 52 (9.68%) |  |
| **Education level (%)** |  |  | <0.001 |
| Less than High school | 62 (7.54%) | 74 (13.78%) |  |
| High school graduate/GED or equivalent | 158 (19.22%) | 133 (24.77%) |  |
| College or above | 602 (73.24%) | 330 (61.45%) |  |
| **Marital status (%)** |  |  | <0.001 |
| Married/living as married | 537 (65.33%) | 303 (56.42%) |  |
| Single/divorced/widowed/ never married | 285 (34.67%) | 234 (43.58%) |  |
| **Body mass index [kg/m2, medium (IQR)]** | 27.90 (24.70-31.70) | 29.30 (25.30-33.80) | <0.001 |
| **PIR [medium (IQR)]** | 3.52 (1.79-5.00) | 2.44 (1.26-4.15) | <0.001 |
| **Drinking status (%)** |  |  | 0.009 |
| No | 204 (24.82%) | 168 (31.28%) |  |
| Yes | 618 (75.18%) | 369 (68.72%) |  |
| **Smoking status (%)** |  |  | 0.551 |
| No | 403 (49.03%) | 274 (51.02%) |  |
| Now | 320 (38.93%) | 208 (38.73%) |  |
| Former | 99 (12.04%) | 55 (10.24%) |  |
| **Work activity (%)** |  |  | 0.107 |
| Vigorous | 123 (14.96%) | 66 (12.29%) |  |
| Moderate | 189 (22.99%) | 108 (20.11%) |  |
| Other | 510 (62.04%) | 363 (67.60%) |  |
| **Recreational activity (%)** |  |  | 0.001 |
| Vigorous | 134 (16.30%) | 57 (10.61%) |  |
| Moderate | 312 (37.96%) | 188 (35.01%) |  |
| Other | 376 (45.74%) | 292 (54.38%) |  |
| **Hypertension (%)** |  |  | <0.001 |
| No | 315 (38.32%) | 151 (28.12%) |  |
| Yes | 507 (61.68%) | 386 (71.88%) |  |
| **Diabetes (%)** |  |  | 0.006 |
| No | 660 (80.29%) | 397 (73.93%) |  |
| Yes | 162 (19.71%) | 140 (26.07%) |  |
| **Total Word Recall [medium (IQR)]** | 29.00 (26.00-33.00) | 28.00 (25.00-31.00) | <0.001 |
| **Animal Fluency Score [medium (IQR)]** | 20.00 (17.00-23.00) | 19.00 (16.00-22.00) | <0.001 |
| **Digit Symbol Score [medium (IQR)]** | 59.00 (50.00-68.00) | (55.00 (46.00-63.00) | <0.001 |
| **PHQ-9 Score [medium (IQR)]** | 1.00 (0.00-3.00) | 2.00 (0.00-5.00) | <0.001 |
| **Hemoglobin [g/L, medium (IQR)]** | 4.20 (4.10-4.40) | 4.20 (4.00-4.40) | 0.005 |
| **Albumin [g/L, medium (IQR)]** | 14.00 (13.20-14.80) | 13.80 (12.90-14.60) | 0.008 |

Data are median (P25, P75) or numbers and percentages.

P value: Kruskal-Wallis rank sum test for continuous variables, Fisher exact test for categorical variables with expects < 10.

**Supplement Table 2.** Multiple regression analysis of the association of cognitive function scores with the risk of nocturia in non-cognitive impairment.

| **Exposure** | **Non-adjusted** | **Adjust I** | **Adjust II** |
| --- | --- | --- | --- |
| **Total Word Recall Score** | **0.9558 (0.9329, 0.9793) 0.000269** | **0.9612 (0.9365, 0.9866) 0.002884** | **0.9603 (0.9353, 0.9859) 0.002586** |
| **Animal Fluency Score** | **0.9597 (0.9378, 0.9820) 0.000453** | 0.9785 (0.9542, 1.0033) 0.088905 | 0.9832 (0.9584, 1.0086) 0.191920 |
| **Digit Symbol Score** | **0.9704 (0.9617, 0.9793) <0.000001** | **0.9872 (0.9767, 0.9978) 0.018292** | **0.9878 (0.9770, 0.9987) 0.027799** |

Model I was adjusted for gender, age, race, education level, marital status, and poverty-to-income ratio.

Model II was adjusted for gender, age, race, education level, marital status, and poverty-to-income ratio, body mass index, hypertension, diabetes, smoking status, alcohol drinking status, work activities, and recreational activities.
